# Ear Problems Are Associated With Traumatic Dental Injuries Among Australian‐Indigenous‐Children

**DOI:** 10.1111/edt.12988

**Published:** 2024-09-20

**Authors:** Xiangqun Ju, Gloria Mejia, Joanne Hedges, Lisa M. Jamieson

**Affiliations:** ^1^ Australia Research Centre of Public Oral Health, Adelaide Dental School University of Adelaide Adelaide Australia

**Keywords:** ear problems, indigenous children, traumatic dental injuries

## Abstract

**Background/Aim:**

The association between ear problems and traumatic dental injuries (TDIs) has not been examined in longitudinal cohort studies. The study aimed to estimate the effect of ear problems on TDIs in primary and permanent teeth among Australian Indigenous children.

**Methods:**

The Longitudinal Study of Indigenous Children is a study of two representative cohorts of Indigenous Australian children, aged from 6 months to 2 years (baby cohort) or from 3.5 to 5 years (child cohort) at baseline (2008). The children's mother/primary carer undertook a face‐to‐face interview in 2008, repeated annually for the next 9 years. Ear problems included runny ears, perforated eardrum, total deafness, deaf in one ear, hearing loss/partially deaf, and other ear problems. TDIs were teeth and oral soft and hard tissue injuries. Multivariate survival analysis using Cox proportional regression models estimated hazards ratio (HR) were used in the analysis.

**Results:**

A total of 870 from baby cohort and 668 from child cohort Indigenous children, who had no TDIs at baseline were included in the analysis. The prevalence of TDIs was 9.2%, 11.1%, and 6.6% in the total, baby, and child cohorts, respectively. Multivariable models for TDIs indicate children with ear problems had nearly four times (total: HR = 3.72, 95% CI: 1.82–6.77), five times (baby cohort: HR = 4.76, 95% CI: 1.59–11.63), and more than 15 times (child cohort: HR = 16.2, 95% CI: 4.78–49.28) the average hazard over time, than those without ear problems. After adjusting for all covariates, children with ear problems had more than 22 times (HR = 22.03, 95% CI: 4.50–87.07) TDIs than those without ear problems in the child cohort. Mothers/primary carers with lower educational level was positively associated with the incidence of TDIs.

**Conclusion:**

Ear problems were a risk indicator for the increased incidence of TDIs in two large cohorts of Indigenous Australian children. Mothers/primary carers' educational level was a significant risk factor for TDIs.

## 1 Introduction

Traumatic dental injuries (TDIs) are injuries to the teeth and/or other soft and hard tissues within and around the vicinity of the mouth and oral cavity, which the annual incidence of dental trauma globally was 4.5%, with 22.7% primary teeth prevalence (children and toddlers) and 15.2% permanent teeth prevalence (adolescents and adults) [1, 2]. TDIs are the fifth most prevalent disease or injury after dental caries, tension‐type headache, iron‐deficiency anemia, age‐related, and other hearing loss [2, 3]. In Australia, the prevalence of TDIs has been reported to be 6% [4, 5].

Traumatic dental injury is an emergency oral health problem in both children and adolescents, resulting from minor tooth fracture to extensive dentoalveolar damage [6]. Luxation injuries are the most common TDIs in primary dentition, whereas crown fractures are more commonly reported for the permanent teeth [6]. The most frequent causes of TDIs are accidental falls at the global and Australian level. Other causes include sport, bicycling, and traffic accidents [1, 7]. Boys experienced significantly more dental injuries to the permanent teeth than girls [7]. TDIs not only lead to tooth lesions causing aesthetic problems but also have social, psychological, and therapeutic impacts. For these reasons, the negative effect, on oral health‐related quality of life (OHRQoL) has been recognized [8].

Indigenous Australians are people who identify as being of Aboriginal and/or Torres Strait Islander descent. Indigenous children, an estimated 278,000, represent more than one‐third of the Indigenous population (34%) and make up 5.9% of the total child population in Australia [9]. Indigenous children suffer from more caries, gingivitis, and tooth loss than non‐Indigenous children [10, 11, 12]. TDIs are one of the risk factors contributing to tooth loss because tooth extraction (nearly 50%) was the most frequent treatment of TDIs in the primary teeth [8].

Ear problems, such as “otitis media with effusion (OME)” or “glue ear”—an infection of the external ear in children, are considered a common cause of balance instabilities and movement disorganization [13, 14], which may cause falls and lead to TDIs. However, the long‐term effect of ear problems on TDIs has not been investigated. The aim of this study was to estimate the effect of ear problems on TDIs in primary and permanent teeth among Australian Indigenous Children.

## 2 Methods

### 2.1 Study Design and Sample Size

**FIGURE 1:**
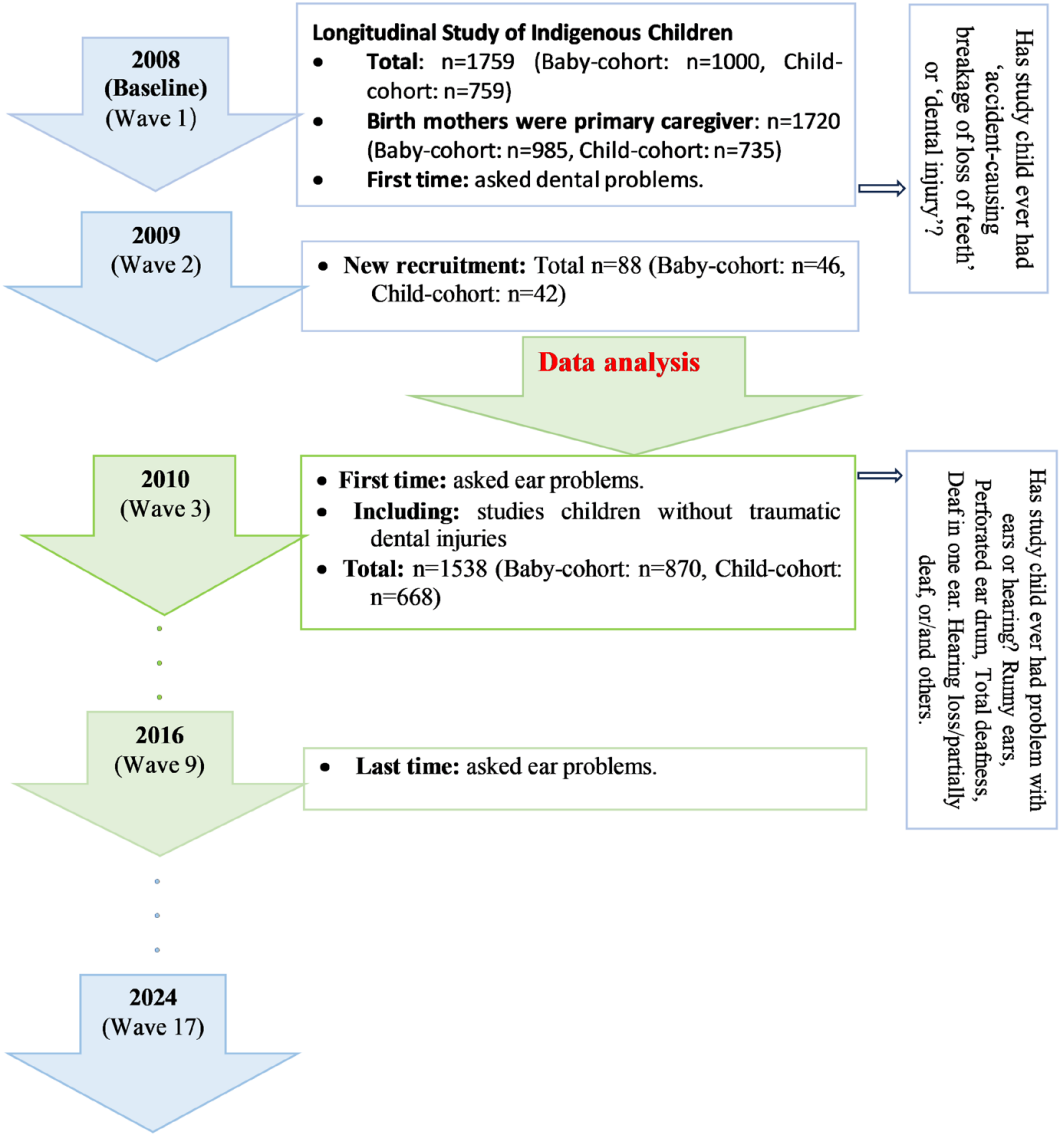
Flow chart of the Longitudinal Study of Indigenous Children (LSIC).

Data were obtained from the Longitudinal Study of Indigenous Children (LSIC) [15]. LSIC is a longitudinal study of two representative cohorts of Indigenous Australian children, aged from 6 months to 2 years (baby cohort) or from 3.5 to 5 years (child cohort) at baseline (2008), and annual follow‐ups until 2024 (see Figure 1).

### 2.2 Data Collection

The child's mother/primary caregiver consented to and undertook a face‐to‐face interview at baseline in 2008 and repeated annually for the next 16 years. New participants were added to the study in 2009. All data were self‐report, with questionnaires including household demographics, and child health (including dental health and other health conditions, such as ears, eyes, and disability status).

### 2.3 Variables

The outcome was TDIs. This variable was from the question: Has the study child ever had “accident‐causing breakage of loss of teeth” or “dental injury”? This variable was classified as “yes” or “no.” Survival times were the age (months) of the first traumatic dental injury.

The exposure variable was ear problems, and was from the question “Has the study child ever had a problem with ears or hearing? (1) runny ears, (2) perforated eardrum, (3) total deafness, (4) deaf in one ear, (5) hearing loss/partially deaf, and (6) others.” This variable was grouped “yes” or “no.” The question was first asked at wave 3 (2010) and was not asked again after wave 9 follow‐up (2016). Therefore, the study used data from baseline (2008) to wave 9 follow‐ups (2016) and study children with TDIs before wave 3 were excluded in data analysis.

Covariates included a study child's and mother/primary caregiver's baseline characteristics. Children's variables included sex (“boy” vs. “girl”), number of birth/children in the family (“1” or “≥ 2”), birth order (“1–2,” “3,” or “≥4”), and other general health condition: eye problems and disability. Eye problem was from the question: “Has the study child ever had any problems with eyes or eyesight? (1) lazy eye, (2) eye infection, (3) cataracts, (4) glaucoma, and (5) totally/partially blind in one or both eyes and was dichotomised into ‘yes’ or ‘no’.” Disability was from the question: “Has the study child had a disability?” (1) intellectual, (2) specific learning, (3) autism spectrum disorder, (4) physical, (5) acquired brain injury, (6) neurological, (7) speech, (8) psychiatric, and (9) other and dichotomised into “yes” or “no.” Mother/ primary caregiver's baseline characteristics included resident location (“regional areas” vs. “major city”) and Socio‐Economic Indexes for Areas (SEIFA) scores which were classified into three approximately equal tertiles (“low,” “medium,” or “high”), and the highest education level (“≤9 years,” “10 years,” “11–12 years,” or “>12 years”).

### 2.4 Statistical Analysis

The analysis began with the computation of univariate statistics describing the frequency and percentage of sample characteristics, and associated prevalence of TDIs, along with 95% confidence intervals (CI) by baby and child cohorts. The mean survival times and survival probability were also estimated for both cohorts. Multivariable survival analysis using Cox proportional regression of time to TDIs models estimated hazard ratios (HR). We started with the crude model (Model 1) by computing univariate Cox analysis for relationships of ear problems and TDIs; the study child's eye and disability conditions were entered in Model 2; children's other characteristics were added in Model 3, and the full model (Model 4) comprised all covariables (adding mothers' characteristics).

Data analyses were performed using SAS statistical software (SAS 9.4, SAS Institute Inc., Cary, NC, USA).

## 3 Results

From 2008 to 2016, there were 1759 study children (1000 from baby cohort and 759 from child cohort), yielding 1720 (97.8%) children whose primary caregiver was their birth mother and who completed the face‐to‐face interview. In total, 1538 of 1720 study children had no dental injury at baseline and second year's follow‐up (870 from baby cohort and 668 from child cohort). The age range of the baby cohort was 8.5–10 years, and the child cohort was 11.5–13 years.

Table 1 presents the sample characteristics, including primary caregiver's characteristics, and study child's birth characteristics and health conditions. A higher proportion were the first‐born child (more than 98%) and one to two of birth order in the family among total, baby and kid cohorts' children. A high proportion did not have ear and eye problems or disabilities among the total sample (94.8%, 98.0%, and 99.3%), baby cohort (94.1%, 97.5%, and 99.2%), and child cohort (95.7%, 98.8%, and 99.6%), respectively. A high proportion of mothers had low SEIFA scores (about 45%) and had an education level >12 years (around 25%) in total and both baby and child cohorts, respectively.

**TABLE 1:** Sample characteristics and prevalence of TDIs among Indigenous children.

	All (*n* = 1538)			Baby cohort (*n* = 870)			Child cohort (*n* = 668)		
	*N*	Percentage (95% CI)	Prevalence of TDI (95% CI)	*N*	Percentage (95% CI)	Prevalence of TDI (95% CI)	*N*	Percentage (95% CI)	Prevalence of TDI (95% CI)
**Total**	1538	100	9.2 (7.7–10.6)	870	56.6 (54.1–59.0)	11.1 (9.1–13.2)		43.4 (41.0–45.9)	6.6 (4.7–8.5)
**Child's characteristics**									
Ear problem									
Yes	80	5.2 (4.1–6.3)	87.5 (80.2–94.8)	51	5.9 (4.3–7.4)	90.2 (82.0–98.4)	29	4.3 (2.8–5.9)	82.8 (69.0–96.5)
No	1458	94.8 (93.7–95.9)	4.9 (3.8–6.0)	819	94.1 (92.6–95.7)	6.2 (4.6–7.9)	639	95.7 (94.1–97.2)	3.1 (1.8–4.5)
Eye problem									
Yes	30	2.0 (1.3–2.6)	90.0 (79.3–100.)	22	2.5 (1.5–3.6)	95.5 (86.7–100)	8	1.2 (0.4–2.0)	75.0 (44.9–100)
No	1508	98.0 (97.4–98.7)	7.6 (6.2–8.9)	848	97.5 (96.4–98.5)	9.0 (7.0–10.9)	660	98.8 (98.0–99.6)	5.8 (4.0–7.5)
Disabilities									
Yes	10	0.7 (0.2–1.1)	90.0 (71.4–100.)	7	0.8 (0.2–1.4)	85.7 (59.7–100)	3	0.4 (0.0–1.0)	100 (100–100)
No	1528	99.3 (98.9–99.8)	8.6 (7.2–10.0)	863	99.2 (98.6–99.8)	10.5 (8.5–12.6)	665	99.6 (99.0–100)	6.2 (4.3–8.0)
Gender									
Boy	781	50.8 (48.3–53.3)	9.9 (7.8–12.0)	442	50.8 (47.5–54.1)	11.1 (8.2–14.0)	339	50.7 (46.9–54.5)	8.3 (5.3–11.2)
Girl	757	49.2 (46.7–51.7)	8.5 (6.5–10.4)	428	49.2 (45.9–52.5)	11.2 (8.2–14.2)	329	49.3 (45.5–53.1)	4.9 (2.5–7.2)
No. of birth									
≥2	27	1.9 (1.2–2.6)	0.0 (0.0–0.0)	15	1.8 (0.9–2.7)	0.0 (0.0–0.0)	12	1.9 (0.8–3.0)	0.0 (0.0–0.0)
1	1425	98.1 (97.4–98.8)	9.4 (7.9–10.9)	805	98.2 (97.3–99.1)	11.6 (9.3–13.8)	620	98.1 (97.0–99.2)	6.6 (4.7–8.6)
Birth order									
≥4	275	25.9 (23.3–28.6)	9.5 (6.0–12.9)	176	28.3 (24.7–31.8)	12.5 (7.6–17.4)	99	22.6 (18.6–26.5)	4.0 (0.1–7.9)
3	212	20.0 (17.6–22.4)	14.6 (9.9–19.4)	129	20.7 (17.5–23.9)	15.5 (9.2–21.8)	83	18.9 (15.2–22.6)	13.3 (5.9–20.6)
1–2	574	54.1 (51.1–57.1)	10.6 (8.1–13.2)	317	51.0 (47.0–54.9)	13.2 (9.5–17.0)	257	58.5 (53.9–63.2)	7.4 (4.2–10.6)
**Mother's characteristics**									
Location									
Regional areas	743	48.5 (46.0–51.0)	7.7 (5.8–9.6)	418	48.3 (45.0–51.7)	10.5 (7.6–13.5)	325	48.7 (44.9–52.5)	4.0 (1.9–6.1)
Major city	790	51.5 (49.0–54.0)	10.5 (8.4–12.6)	447	51.7 (48.3–55.0)	11.6 (8.7–14.6)	343	51.3 (47.5–55.1)	9.0 (6.0–12.1)
SEIFAA									
Low	697	45.9 (43.4–48.5)	8.6 (6.5–10.7)	394	46.0 (42.6–49.3)	9.9 (6.9–12.9)	303	45.9 (42.1–49.7)	6.9 (4.1–9.8)
Medium	501	33.0 (30.7–35.4)	10.8 (8.1–13.5)	289	33.7 (30.6–36.9)	13.8 (9.9–17.8)	212	32.1 (28.5–35.7)	6.6 (3.3–10.0)
High	319	21.0 (19.0–23.1)	8.2 (5.1–11.2)	174	20.3 (17.6–23.0)	9.8 (5.3–14.2)	145	22.0 (18.8–25.1)	6.2 (2.3–10.1)
Education level									
≤9 years	215	16.5 (14.5–18.5)	8.8 (5.0–12.6)	129	17.4 (14.7–20.1)	10.1 (4.9–15.3)	86	15.3 (12.3–18.3)	7.0 (1.6–12.4)
10 years	240	26.1 (23.7–28.4)	10.0 (6.8–13.2)	206	27.8 (24.5–31.00	11.2 (6.9–15.5)	134	23.8 (20.3–27.3)	8.2 (3.5–12.9)
11–12 years	432	33.1 (30.5–35.7)	10.4 (7.5–13.3)	234	31.5 (28.2–34.9)	15.0 (10.4–19.5)	198	35.2 (31.2–39.1)	5.1 (2.0–8.1)
>12 years	318	24.4 (22.0–26.7)	10.4 (7.0–13.7)	173	23.3 (20.3–26.4)	11.0 (6.3–15.7)	145	25.8 (22.1–29.4)	9.7 (4.8–14.5)

Abbreviations: SEIFA, Socio‐Economic Indexes for Areas; TDIs, traumatic dental injuries.

The prevalence of TDIs was 9.2%, 11.1%, and 6.6% in the total, baby, and child cohorts, respectively. The prevalence of TDIs in those without ear and eye problems and disability were 87.5%, 90.0%, and 90.0%, respectively, among the total cohort, and 90.2%, 95.5%, and 85.7%, respectively, among the baby cohort, and 82.8%, 75.0%, and 100%, respectively, among the child cohort (Table 1).

**FIGURE 2:**
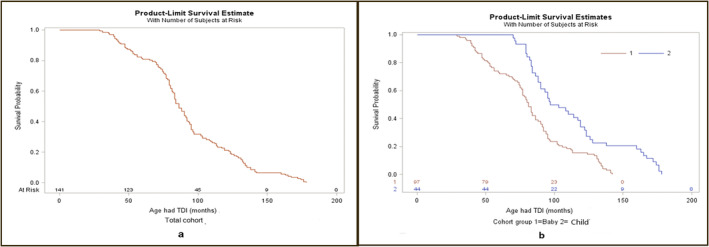
Plots of Kaplan–Meier product limit estimate of survival probability in Indigenous Australian children.

The average survival times of TDIs were 91.7, 82.4, and 112.4 months in total, baby, and child cohorts, respectively (Table 2). Figure 2 shows the survival curves for total (Figure 2a) and baby and child cohorts (Figure 2b). In Figure 2a, it appears the probability of surviving beyond 150 months is a little <0.1, which confirms that the probability of surviving 150 months or fewer is a little more than 0.9. In Figure 2b, the blue curves (baby cohort) show that the probability of a TDI occurring (TDI survival) beyond 100 months is a little <0.2, which is confirmed that the probability of TDI survival at 100 months or fewer is a little more than 0.8. The red curves (child cohort) show that the probability of surviving beyond 150 months is a little more than 0.2, which confirms that the probability of surviving 150 months or fewer is a little more than 0.8. The results indicated that the surviving probability was higher in child cohort than was in baby cohort.

**TABLE 2:** Mean survival times (months) had TDIs among Indigenous children.

	Minimum	Maximum	Mean (95% CI)
All			
All	29	178	91.7 (86.0–97.5)
Baby cohort	29	142	82.4 (76.3–88.5)
Child cohort	70	178	112.4 (102.0–122.8)
Had ear problem			
All	29	138	85.1 (79.4–90.9)
Baby cohort	29	113	76.7 (70.4–82.9)
Child cohort	70	138	101.4 (92.8–110.1)
No ear problem			
All	31	178	98.3 (88.5–108.0)
Baby cohort	31	142	87.5 (77.5–97.6)
Child cohort	79	178	125.6 (105.7–145.4)

Abbreviation: TDIs, traumatic dental injuries.

Tables 3, 4, 5 present multivariable Cox regression models for TDIs. Children with ear problems had nearly four times (total: HR = 3.72, 95% CI: 1.82–6.77), four times (baby cohort: HR = 4.76, 95% CI: 1.59–11.63), and more than 15 times (child cohort: HR = 16.2, 95% CI: 4.78–49.28) the average hazard over time, than those without ear problems. After adjusting for all covariates, children with ear problems had more than 22 times (HR = 22.03, 95% CI: 4.50–87.07) TDIs than those without ear problems in the child cohort only (Table 3). Mothers/primary carers with the lowest educational level (≤9 years) had 1.3 and 1.5 times higher incidence of TDIs than mothers with the highest education level (>12 years) in total and child cohort, respectively (Tables 3 and 5).

**TABLE 3:** Cox regression models for TDIs among Indigenous Australia children (hazard ratio [HR] and 95% CI).

	Model 1	Model 2	Model 3	Model 4
	HR (95% CI)	HR (95% CI)	HR (95% CI)	HR (95% CI)
Ear problem				
Yes	3.72 (1.82–6.77)	3.17 (1.32–6.39)	2.70 (0.91–6.21)	2.68 (0.88–6.31)
No	Ref	Ref	Ref	Ref
Eye problem				
Yes	4.70 (1.14–13.0)	1.76 (0.38–6.10)	1.48 (0.21–6.56)	1.63 (0.23–7.65)
No	Ref	Ref	Ref	Ref
Disabilities				
Yes	4.71 (0.27–21.55)	1.58 (0.09–8.51)	2.18 (0.12–12.9)	4.23 (0.22–27.57)
No		Ref	Ref	Ref
Gender				
Boy			0.99 (0.86–1.13)	1.02 (0.88–1.18)
Girl			Ref	Ref
No. of birth				
≥2			0.96 (0.56–1.52)	0.97 (0.56–1.54)
1			Ref	Ref
Birth order				
≥4			1.12 (0.95–1.31)	1.11 (0.94–1.32)
3			0.96 (0.80–1.15)	0.94 (0.78–1.14)
1–2			Ref	Ref
Location				
Regional areas				0.93 (0.80–1.08)
Major city				Ref
SEIFA				
Low				1.02 (0.84–1.23)
Medium				0.97 (0.80–1.18)
High				Ref
Education level				
≤9 years				1.28 (1.01–1.62)
10 years				1.09 (0.89–1.33)
11–12 years				1.06 (0.87–1.28)
>12 years				Ref

*Note:* Model 1: univariate cox analysis for relationships of ear problems and TDIs; Model 2: plus adjusting for study child's eye and disability conditions; Model 3: Model 2 plus adjusting for children's other characteristics; Model 4 (Full model): model 3 plus adjusting for mothers' characteristics.

Abbreviations: SEIFA, Socio‐Economic Indexes for Areas; TDIs, traumatic dental injuries.

**TABLE 4:** Cox regression models for TDIs among baby cohort of Indigenous Australia children (hazard ratio [HR] and 95% CI).

	Model 1	Model 2	Model 3	Model 4
	HR (95% CI)	HR (95% CI)	HR (95% CI)	HR (95% CI)
Ear problem				
Yes	4.76 (1.59–11.63)	4.86 (1.30–13.30)	2.55 (0.35–10.36)	2.61 (0.33–11.50)
No	Ref	Ref	Ref	Ref
Eye problem				
Yes	2.10 (0.12–10.20)	0.56 (0.03–3.65)	1.03 (0.05–8.77)	1.32 (0.06–13.59)
No		Ref	Ref	Ref
Disabilities				
Yes	8.6 (0.48–42.7)	2.51 (0.13–16.63)	3.87 (0.18–34.0)	4.50 (0.20–47.93)
No	Ref	Ref	Ref	Ref
Gender				
Boy			1.07 (0.89–1.28)	1.09 (0.89–1.33)
Girl			Ref	Ref
No. of birth				
≥2			0.86 (0.36–1.68)	0.88 (0.37–1.74)
1			Ref	Ref
Birth order				
≥4			1.06 (0.85–1.30)	1.08 (0.86–1.36)
3			0.82 (0.64–1.04)	0.82 (0.63–1.06)
1–2			Ref	Ref
Location				
Regional areas				0.92 (0.75–1.13)
Major city				Ref
SEIFA				
Low				0.99 (0.77–1.28)
Medium				0.96 (0.75–1.25)
High				Ref
Education level				
≤9 years				1.07 (0.78–1.45)
10 years				1.26 (0.96–1.66)
11–12 years				1.29 (0.98–1.68)
>12 years				Ref

*Note:* Model 1: univariate cox analysis for relationships of ear problems and TDIs; Model 2: plus adjusting for study child's eye and disability conditions; Model 3: Model 2 plus adjusting for children's other characteristics; Model 4 (Full model): model 3 plus adjusting for mothers' characteristics.

Abbreviations: SEIFA, Socio‐Economic Indexes for Areas; TDIs, traumatic dental injuries.

**TABLE 5:** Cox regression models for TDIs among child cohort of Indigenous Australian children (hazard ratio [HR] and 95% CI).

	Model 1	Model 2	Model 3	Model 4
	HR (95% CI)	HR (95% CI)	HR (95% CI)	HR (95% CI)
Ear problem				
Yes	16.2 (4.78–49.28)	13.83 (3.09–47.36)	23.37 (4.77–92.27)	22.03 (4.50–87.07)
No	Ref	Ref	Ref	Ref
Eye problem				
Yes	18.5 (2.78–73.80)	2.24 (0.30–13.03)	1.01 (0.05–7.85)	0.95 (0.05–7.44)
No	Ref	Ref	Ref	Ref
Disabilities				
Yes		0.0 (0.0–8.75)	0.0 (0.0–8.54)	0.0 (0.0–19.95)
No		Ref	Ref	Ref
Gender				
Boy			0.95 (0.77–1.17)	1.04 (0.83–1.30)
Girl			Ref	Ref
No. of birth				
≥2			1.18 (0.56–2.17)	1.12 (0.53–2.07)
1			Ref	Ref
Birth order				
≥4			0.95 (0.73–1.22)	0.95 (0.72–1.24)
3			0.82 (0.61–1.09)	0.82 (0.60–1.10)
1–2			Ref	Ref
Location				
Regional areas				0.94 (0.74–1.20)
Major city				Ref
SEIFA				
Low				0.99 (0.75–1.32)
Medium				0.87 (0.65–1.17)
High				Ref
Education level				
≤9 years				1.53 (1.04–2.23)
10 years				1.02 (0.75–1.40)
11–12 years				1.14 (0.86–1.52)
>12 years				Ref

*Note:* Model 1: univariate cox analysis for relationships of ear problems and TDIs; Model 2: plus adjusting for study child's eye and disability conditions; Model 3: Model 2 plus adjusting for children's other characteristics; Model 4 (Full model): model 3 plus adjusting for mothers' characteristics.

Abbreviations: SEIFA, Socio‐Economic Indexes for Areas; TDIs, traumatic dental injuries.

## 4 Discussion

To the best of our knowledge, this study is the first to investigate associations between ear problems and TDIs among Indigenous Australian children. Our findings indicated that the prevalence of TDIs was nearly two times higher among younger children (baby cohort) than older children (child cohort). Children with ear problems had a higher risk of TDIs than those without ear problems in both baby and child cohorts, in particular children in child cohort up to more than 20 times after adjusting for other health problems and covariates. Low education level among carers was also positively associated with TDIs.

Our findings demonstrated that ear problems are important risk factors for TDIs. One possible mechanism is that ear infections, such as chronic OME, have been identified as a principal cause of balance instabilities and vertigo in children, resulting in falls and TDIs [13]. If chronic OME lasts for more than 3 months, about 25% of children would lose their hearing [14]. Hearing loss is another risk factor for falls, due to its effects on balance [16]. Hearing loss makes people less aware of their surroundings, decreases spatial awareness, or causes the brain to use more resources for hearing and interpreting speech and sound, resulting in body imbalance and falls.

Our findings were similar with previous studies [8, 17] that dental injuries were more frequent among 8‐ to 10‐year‐old children. TDIs are one of the most serious oral health problems among children, not only causing tooth lesions and even loss, leading to possible negative outcomes (such as chewing problems and pronunciation); but also affecting social and psychological health, such as difficulties with social interaction, avoidance of smiling and laughing and not talking to other children [8, 18]. Ultimately, OHRQoL deteriorates. In addition, our study found that children's TDIs associated with their mothers' low educational attainment. This may be because carers with less educational attainment may have low general and/or oral health literacy [19], resulting in delay in obtaining dental emergency care [17]. Also, children whose mother with low socioeconomic status (SES) have more ear problems (such as ear infections and hearing loss), therefore they have more TDIs [20, 21]. At the same time, it is inevitable that racial discrimination may play an important role in mothers/primary carers not going to a doctor when their children suffered from ear problems, or not accessing a dentist at the time of TDIs due to distrust in public health/dental services [22], eventually, ear problems get worse or more TDIs occur, lead to more tooth loss or extractions. Therefore, dental first aid training for mothers/caregivers is essential, as well as letting them know to obtain emergency dental care immediately after sustaining TDIs.

The strengths of the study included: (1) being the first time in Australia to investigate the association between ear problems and TDIs and (2) longitudinal cohort study design with large sample size and different cohorts were estimated and compared. The latter enriched and increased the reliability of our findings and provided important evidence to clinicians and policymakers. Limitation of the study was the lack of clinical examination data. Self‐reported data collection relies on recall so this may be over‐ or under‐estimated. However, the longitudinal study design with annually asking the same questions might reduce this recall bias.

## 5 Conclusion

Ear problems were a risk indicator for the increased incidence of TDIs in two large cohorts of Indigenous Australian children. Low educational attainment of primary carers was an additional risk factor for TDIs.

## Author Contributions

Conceptualization, X.J., G.M., and L.M.J.; methodology, X.J., G.M., and L.M.J.; software, X.J., and G.M.; validation, X.J., and L.M.J.; formal analysis, X.J., and G.M.; data curation, X.J., and G.M.; writing – original draft preparation, X.J.; writing – review and editing, X.J., G.M., J.H., and L.M.J.; visualization, L.M.J.; supervision, L.M.J.; project administration, X.J. All authors have read and agreed to the published version of the manuscript.

## Ethics Statement

Ethical approval was obtained from the Australian Government Department of Health Departmental Ethics Committee, the nominated Human Research Ethics Committee (EC000106) for LSIC.

## Consent

All participants were provided with an information sheet outlining the study objectives and signed an informed consent form.

## Conflicts of Interest

The authors declare no conflicts of interest.

## Data Availability

The datasets generated and/or analyzed during the current study are not publicly available due to privacy issues of the participants. Data is available from the corresponding author on reasonable request.
